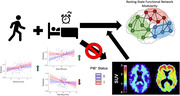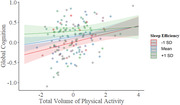# Sleep and Physical Activity are Associated with Cognition and Resting‐State Functional Imaging Measures of Network Segregation in Non‐demented Older Adults

**DOI:** 10.1002/alz.093765

**Published:** 2025-01-09

**Authors:** Daniel Callow

**Affiliations:** ^1^ Johns Hopkins University School of Medicine, Baltimore, MD USA

## Abstract

**Background:**

Greater physical activity and better sleep are associated with reduced risk of cognitive decline and dementia among older adults, but little is known about their associations with measures of brain function and neuropathology.

**Method:**

This study investigated potential independent and interactive cross‐sectional relationships between actigraphy‐estimated total volume of physical activity (TVPA) and sleep patterns [i.e., total sleep time (TST), sleep efficiency (SE)] with cognitive performance (n = 157). In a subset of participants, the physical activity and sleep measures were also evaluated in relationship to resting‐state functional magnetic resonance imaging (rs‐fMRI) measures of large‐scale network connectivity, and positron emission tomography (PET) measures of amyloid‐ß (n = 135). Participants were part of the BIOCARD study including 136 cognitively normal individuals and 21 participants with mild cognitive impairment (mean age = 71.7 years). Using linear regression analyses, we assessed the association between TVPA, TST, and SE with cognition, connectivity within the default‐mode, salience, and fronto‐parietal control networks, and with network modularity, a measure of network segregation.

**Result:**

Greater TVPA and SE each were positively associated with better global cognitive composite scores and executive function. Importantly, a TVPA by SE interaction suggested that adults with the poorest SE experienced the greatest benefit from physical activity in relation to cognition. In the imaging subsample, higher TVPA and SE were independently associated with greater network modularity, although the positive relationship of SE with modularity was only present in amyloid‐negative individuals. Additionally, higher TVPA was associated with greater connectivity within the default‐mode network, while greater SE was related to greater connectivity within the salience network. In contrast, longer TST was associated with lower network modularity, particularly among amyloid‐positive individuals, suggesting a relationship between longer sleep duration and greater network disorganization. Physical activity and sleep measures were not associated with amyloid positivity.

**Conclusion:**

These data suggest that greater physical activity levels and more efficient sleep may promote better cognition and more segregated and potentially resilient functional networks, as well as greater functional connectivity within specific large‐scale networks. Additionally, the relationship between sleep and functional networks connectivity may depend on amyloid status while associations with physical activity may be independent of amyloid status.